# Thyroid-Stimulating Hormone Inhibits Insulin Receptor Substrate-1 Expression and Tyrosyl Phosphorylation in 3T3-L1 Adipocytes by Increasing NF-*κ*B DNA-Binding Activity

**DOI:** 10.1155/2022/7553670

**Published:** 2022-03-14

**Authors:** Yajing Zhang, Ling Feng

**Affiliations:** ^1^NHC Key Laboratory of Hormones and Development, Tianjin Key Laboratory of Metabolic Diseases, Chu Hsien-I Memorial Hospital & Tianjin Institute of Endocrinology, Tianjin Medical University, Tianjin 300134, China; ^2^People's Hospital of Guangxi Zhuang Autonomous Region, China

## Abstract

**Background:**

Abundant evidence indicates that thyroid-stimulating hormone (TSH) levels are associated with insulin resistance in adipocytes. However, the potential mechanism of the association remains uncertain. The objective of this study was to determine the potential role of TSH in the suppression of insulin receptor substrate-1 (IRS-1) expression and IRS-1 tyrosyl phosphorylation, which might contribute to insulin resistance.

**Methods:**

Mouse 3T3-L1 preadipocytes were differentiated into adipocytes. After treatment with 0.01, 0.1, and 1.0 mIU/ml bovine TSH, the TNF-*α* concentration in the medium was determined by enzyme-linked immunosorbent assay (ELISA). Nuclear factor-kappa B (NF-*κ*B) DNA-binding activity was quantified by electrophoretic mobility shift assay (EMSA). IRS-1 levels in adipocytes were quantified by Western blotting, and tyrosine phosphorylation was measured by immunoprecipitation.

**Results:**

TSH induced TNF-*α* secretion in a dose-dependent manner. There was a significant positive correlation between NF-*κ*B DNA-binding activity and TNF-*α* secretion. This effect and correlation were weakened by BAY 11-7082 (a nuclear NF-*κ*B inhibitor) and H89 (an inhibitor of cyclic adenosine monophosphate- (cAMP-) dependent protein kinase A (PKA)). Treatment of cultured adipocytes with TSH inhibited insulin-stimulated IRS-1 tyrosyl phosphorylation but promoted TSH-dependent secretion of TNF-*α* and activation of NF-*κ*B DNA-binding activity. The effects of TSH were significantly inhibited by BAY 11-7082 and H89 and were completely blocked by the TNF-*α* antagonist WP9QY.

**Conclusion:**

TSH inhibited IRS-1 protein expression and tyrosyl phosphorylation in 3T3-L1 adipocytes by stimulating TNF-*α* production via promotion of NF-*κ*B DNA-binding activity. TSH might play a pivotal role in the development of insulin resistance.

## 1. Introduction

Diabetes mellitus (DM) is a major chronic disease affecting humans. More than 95% of all cases of diabetes are type 2 DM (T2DM) [1]. The etiology of T2DM is associated with insulin resistance; specifically, insulin resistance in adipose tissue is a prominent T2DM feature. This insulin resistance results from decreased insulin sensitivity in tissues such as adipose, skeletal muscle, and liver tissues. Adipose tissue secretes numerous cytokines (adipokines), such as leptin [2], tumor necrosis factor-alpha (TNF-*α*), and interleukin 6 (IL-6) [3, 4]. The association between adipokines and insulin resistance is well known. It has been reported that TNF-*α* is the key mediator of insulin resistance in obesity because it suppresses insulin activity [5] by potently inhibiting the expression and tyrosyl phosphorylation of insulin receptor substrate-1 (IRS-1, the major insulin-like growth factor-I receptor), which might reduce the ability of IRS-1 to transduce signals in the insulin signaling system [6].

Thyroid-stimulating hormone receptor (TSHR) is expressed not only in thyrocytes but also in adipocytes. Thyroid-stimulating hormone (TSH) may activate TSHR in adipose cells [7, 8]. We previously reported that TSH stimulates TNF-*α* secretion from adipocytes via a cyclic adenosine monophosphate- (cAMP-) protein kinase A- (PKA-) dependent pathway [9]. Recently, increasing clinical evidence of the association between TSH and insulin resistance has been obtained [10–12]. However, a few basic studies on the mechanism and signaling pathways of the association between TSH and insulin resistance have been reported. The present study was designed to assess the changes in NF-*κ*B DNA-binding activity, IRS-1 expression, and IRS-1 tyrosyl phosphorylation in 3T3-L1 adipocytes after treatment with TSH and then to determine the potential role of TSH in the suppression of IRS-1 expression and tyrosyl phosphorylation, which might contribute to insulin resistance.

## 2. Materials and Methods

### 2.1. Cell Culture and Induced Differentiation of Adipocytes

Mouse 3T3-L1 preadipocytes (ATCC, China) were seeded at a density of 1.5 × 10^4^ cells/cm^2^ in Corning polystyrene culture dishes and cultured in Dulbecco's modified Eagle's medium (DMEM) supplemented with 10% calf serum (CS), 100 U/ml penicillin, and 0.1 mg/ml streptomycin (KeyGEN, Nanjing, China) in a humidified 5% CO_2_ incubator at 37°C. The medium was replaced every two days. Upon reaching 60-70% confluence, the cells were seeded for experiments and were passaged a maximum of three times. The 3T3-L1 preadipocytes were differentiated as described previously [9].

The adipocytes were starved overnight in serum-free DMEM and treated with different concentrations of TSH (0.01, 0.1, and 1 mIU/ml) or pretreated with 5 *μ*M BAY 11-7082 (a nuclear factor-kappa B (NF-*κ*B) inhibitor, Biyuntian Institute of Biological Technology, China) or 10 *μ*M H89 (an inhibitor of cAMP-dependent PKA, Sigma, CA, USA) for 15 min. The cells and medium were harvested for enzyme-linked immunosorbent assay (ELISA), electrophoretic mobility shift assay (EMSA), or Western blot analysis.

### 2.2. ELISA

After treatment, the TNF-*α* concentration in the medium was determined using an ELISA kit (R&D Systems) according to the manufacturer's instructions.

### 2.3. Nuclear Extract Preparation

Nuclear extracts were prepared from cells using an NE-PER Nuclear and Cytoplasmic Extraction Reagent Kit according to the manufacturer's directions (KeyGEN, Nanjing, China) with some modifications. After two washes with 10 ml of phosphate-buffered saline (PBS), the cells were harvested into 75-cm^2^ culture flasks and pelleted by centrifugation at 500 × *g* for 3 min at 4°C. Each cell pellet was resuspended by gentle pipetting in 1.3 ml of buffer A (10 mM HEPES-KOH (pH 7.9), 10 mM KCl, 1.5 mM MgCl2, 2 mM EDTA, 1 mM dithiothreitol (DTT), 1 mM phenylmethanesulfonyl fluoride, and 1 *μ*M pepstatin). After centrifugation at 12,000 × *g* for 15 sec at 4°C, the cells were lysed via incubation on ice for 10 min in 100 *μ*l of buffer B and then centrifuged at 16,000 × *g* for 5 min. The pellet was washed with PBS(−), resuspended in 100 *μ*l of buffer C (20 mM HEPES-KOH (pH 7.9), 0.4 M KCl, 2 mM EDTA, 1 mM DTT, 1 mM phenylmethanesulfonyl fluoride, and 1 *μ*M pepstatin) and lysed by freezing and thawing. After centrifugation at 16,000 × *g* for 10 min at 4°C, the supernatant was used as a nuclear extract. The protein concentration in each lysate was measured by bicinchoninic acid (BCA) assay. The extracts were aliquoted and stored at -80°C until analysis by EMSA.

### 2.4. EMSA

EMSA was performed using a Chemiluminescent EMSA Kit (Biyuntian Institute of Biological Technology, China) according to the manufacturer's protocol with double-stranded oligonucleotides encoding the NF-*κ*B consensus sequence (5′-AGT TGA GGG GAC TTT CCC AGG C-3′ and 3′-TCA ACT CCC CTG AAA GGG TCC G-5′), which were end-labeled with biotin (Biyuntian Institute of Biological Technology, China). Nuclear extracts (2.5 *μ*g) were added to 10 *μ*l of binding reagent and incubated for 20 min at room temperature. The bound complexes were resolved by electrophoresis on nondenaturing 6% polyacrylamide gels using 0.5× TBE as the running buffer and assessed by autoradiography. To establish the specificity of the reaction, negative controls without cell extracts were used. In a cold probe competition reaction, 50-fold corresponding unlabeled probe was added for preincubation.

### 2.5. Western Blotting

The procedures used for preparation of cytosolic extracts and Western blot analysis have been described previously [9]. In brief, the protein concentration in each lysate was measured by BCA assay. Equal amounts of protein were separated by SDS-PAGE. After electrophoresis, the proteins were transferred onto nitrocellulose membranes. The membranes were incubated with an anti-IRS-1-pTyr^15^ antibody (1 : 500, Biyuntian Institute of Biological Technology, China) or an anti-*β*-tubulin antibody (1 : 1000, Sungene Biotech Co., Ltd., Tianjin, China). The membranes were then incubated with a peroxidase-conjugated secondary antibody (1 : 5000, Santa Cruz Biotechnology). After rinsing, chemiluminescent detection was performed using an ECL Western blot detection kit followed by exposure and development of X-ray film. The Western blot results were analyzed by densitometry. Where indicated, the membranes were stripped according to the manufacturer's directions before reblotting.

### 2.6. Immunoprecipitation

3T3-L1 adipocytes were lysed in RIPA buffer on ice for 10 min. The cytosolic extracts were prepared by centrifuging the lysates twice at 12,000 rpm for 10 min at 4°C. The cytosolic extracts were incubated with an anti-IRS-1 antibody overnight at 4°C with shaking. The immunocomplexes were captured by adding protein A/G-agarose and gently rotating the samples for 2 h at 4°C. The immunocomplex precipitates were washed with PBS three times, and the immunoprecipitated proteins were subjected to Western blot analysis using an anti-IRS-1 antibody.

### 2.7. Statistical Analyses

Each experiment was performed at least in triplicate. Statistical analyses were performed using Student's *t*-test or analysis of variance (ANOVA). All the data are presented as the mean ± SD, and *P* < 0.05 was considered to indicate significance.

## 3. Results

**Figure 1:**
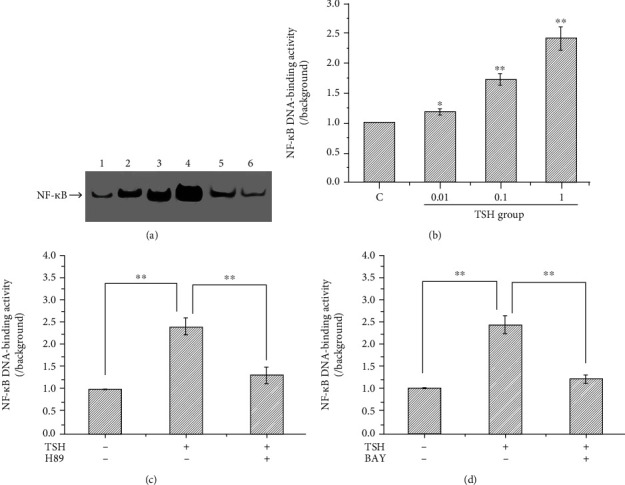
Effect of TSH on the DNA-binding activity of NF-*κ*B in 3T3-L1 adipocytes. (a) Representative autoradiogram showing the NF-*κ*B DNA-binding activity in each group. 1: the control, 2: the TSH concentration of 0.01 mIU/ml, 3: the TSH concentration of 0.1 mIU/ml, 4: the TSH concentration of 1 mIU/ml, 5: after H89; and 6: after BAY 11-7082. (b) NF-*κ*B DNA-binding activity was significantly and dose-dependently upregulated in cells treated with different concentrations of TSH (0.01, 0.1, and 1 mIU/ml) compared with control cells (*P* < 0.05), especially in cells stimulated with 0.1 mIU/ml and 1 mIU/ml TSH (*P* < 0.01). (c) Effects of H89 on NF-*κ*B DNA binding. (d) Effects of BAY 11-7082 on NF-*κ*B DNA-binding. Cells cultured under the same conditions were pretreated with H89 (an inhibitor of cAMP-dependent PKA) or BAY 11-7082 (a nuclear NF-*κ*B inhibitor). NF-*κ*B DNA-binding activity was suppressed after treatment with H89 and BAY 11-7082. The DNA-binding activity of NF-*κ*B was quantified by densitometric analysis. The band intensities were normalized relative to the internal control and background. The number 1 represents the control group; 2, 3, and 4 represent the 0.01, 0.1, and 1 mIU/ml TSH groups, respectively; and 5 and 6 represent the H89- and BAY 11-7082-pretreated groups, respectively. The data are presented as the mean ± SD from three independent experiments (^∗∗^*P* < 0.01 vs. the 1 mIU/ml TSH group).

In a previous study, we detected the I*κ*B*α*/PKAc complex in lysates of 3T3-L1 adipocytes treated with bovine TSH by coimmunoprecipitation and Western blotting [9]. To investigate whether TSH activates NF-*κ*B, we measured the NF-*κ*B DNA-binding activity in 3T3-L1 adipocytes treated with different concentrations of TSH (0.01, 0.1, and 1 mIU/ml) or pretreated with 5 *μ*M BAY 11-7082 (a nuclear NF-*κ*B inhibitor) or 10 *μ*M H89 (an inhibitor of cAMP-dependent PKA). Nuclear extracts were used to analyze NF-*κ*B DNA-binding activity via EMSA. It was found that NF-*κ*B DNA-binding activity was significantly and dose-dependently upregulated in cells treated with different concentrations of TSH (0.01, 0.1, and 1 mIU/ml) compared with control cells, especially in cells stimulated with 0.1 mIU/ml and 1 mIU/ml TSH (*P* < 0.01) (Figures 1(a) and 1(b)). These results indicated that TSH increased NF-*κ*B DNA-binding activity in adipocytes. Pretreatment with H89 significantly suppressed the TSH-dependent activation of the slow-migrating complex (Figures 1(a) and 1(c)). No apparent protein-*κ*Bwt complex was observed when the nuclear extracts prepared from cells pretreated with 5 *μ*M BAY 11-7082 were examined (Figures 1(a) and 1(d)). This result indicated that BAY 11-7082 and H89, especially BAY 11-7082, significantly blocked TSH-stimulated NF-*κ*B activation. This binding reaction was shown to be specific since an excess of unlabeled NF-*κ*B probe decreased the signals of the slow-migrating bands.

**Figure 2:**
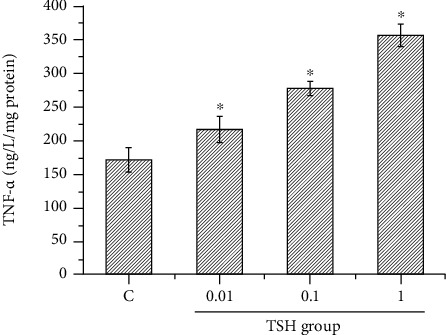
TSH-stimulated 3T3-L1 adipocytes produce TNF-*α*. Differentiated adipocytes were treated with different concentrations of TSH (0.01, 0.1, and 1 mIU/ml) for 4 h, and the TNF-*α* concentration in the medium was measured by ELISA. Increasing doses of TSH stimulated 3T3-L1 adipocytes to secrete TNF-*α*. The data are presented as the mean ± SD (*n* = 3) (*P* < 0.05 for all comparisons).

**Figure 3:**
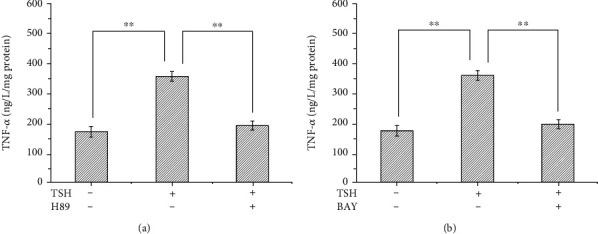
Factors that affect TNF-*α* secretion from 3T3-L1 adipocytes. (a) Pretreatment of cells with the PKA inhibitor H89 (10 *μ*M) for 15 minutes before treatment with 1 mIU/ml bovine TSH decreased TNF-*α* levels. (b) Pretreatment with the nuclear NF-*κ*B inhibitor BAY 11-7082 (5 *μ*M) reduced TSH-stimulated TNF-*α* production. These results suggested that H89 and BAY 11-7082 inhibited the effect of TSH on TNF-*α* secretion. The data demonstrated that TSH stimulated 3T3-L1 adipocytes to secrete TNF-*α* via the cAMP-PKA pathway. The data are presented as the mean ± SD (*n* = 3) (^∗∗^*P* < 0.01).

We have previously reported that TSH stimulates TNF-*α* secretion from adipocytes in a dose-dependent manner by elevating the levels of phospho-NF-*κ*Bp65 Ser276 via the PKA signaling pathway [9]. To investigate the effects of NF-*κ*B DNA-binding activity on TSH-stimulated TNF-*α* secretion, adipocytes were treated with different concentrations of TSH (0.01, 0.1, and 1 mIU/ml) for 4 h. Some cells were pretreated with 10 *μ*M H89 or 5 *μ*M BAY 11-7082 for 15 min and then treated with 1 mIU/ml TSH for 4 h; the TNF-*α* concentration in the medium was measured by ELISA. These experiments showed the same results as our previous study: TNF-*α* secretion increased with increasing doses of TSH (*P* < 0.01 or *P* < 0.05) (Figure 2). H89 and BAY 11-7082 reduced TSH-stimulated TNF-*α* production by ~46% (Figures 3(a) and 3(b)). Previous research has shown that free NF-*κ*B translocates into the nucleus, binds to specific DNA regions, and triggers numerous transcriptional events and cellular responses [13]. Since TNF-*α* transcription is positively regulated by NF-*κ*B, the experimental results indicated that TSH-stimulated NF-*κ*B DNA-binding activity resulted in enhanced TNF-*α* transcriptional activity via the PKA signaling pathway.

**Figure 4:**
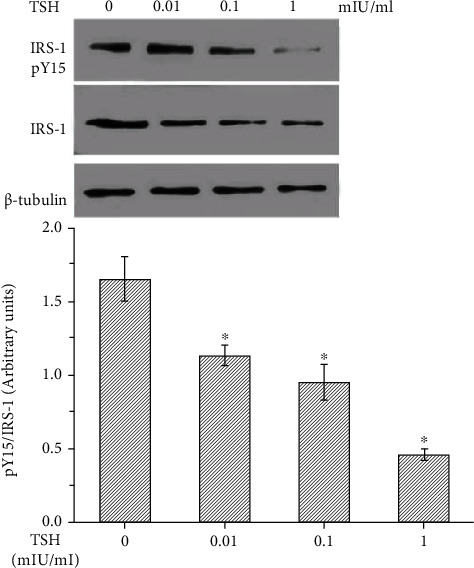
TSH decreased tyrosine phosphorylation of IRS-1 in 3T3-L1 adipocytes. Cells were pretreated with 100 nM insulin for 10 min and then treated with different concentrations of TSH (0.01, 0.1, and 1 mIU/ml) for 24 h. IRS-1 levels in adipocytes were quantified by Western blotting, and tyrosine phosphorylation was measured by immunoprecipitation. The relative expression levels of IRS-1 and IRS-1-tyr in each treatment group were calculated using *β*-tubulin as the standard. Treatment with various concentrations of bovine TSH (0.01, 0.1, and 1 mIU/ml) increased the ratio of phosphorylated IRS-1 to total IRS-1 in a dose-dependent manner in 3T3-L1 adipocytes. These data indicated that TSH suppressed IRS-1 tyrosyl phosphorylation in 3T3-L1 adipocytes (*P* < 0.05 for all comparisons).

**Figure 5:**
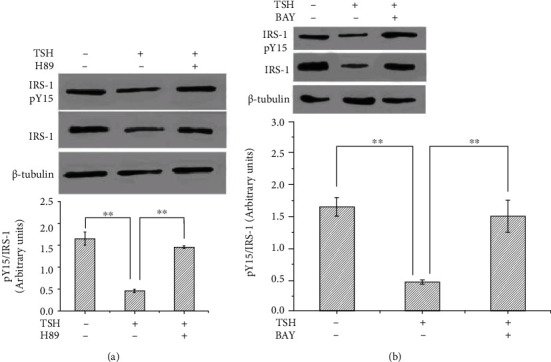
BAY 11-7082 and H89 inhibited the TSH-mediated downregulation of tyrosine phosphorylation of IRS-1 in 3T3-L1 adipocytes. (a) After pretreatment with 10 *μ*M H89 for 15 minutes and treatment with 1 mIU/ml bovine TSH, phosphorylated IRS-1 levels were unchanged in 3T3-L1 adipocytes. (b) Pretreatment of cells with BAY 11-7082 inhibited the TSH-dependent increases in phosphorylated IRS-1 levels. The data are presented as the mean ± SD (*n* = 3) (^∗^*P* < 0.05,  ^∗∗^*P* < 0.01).

Previously, Shen et al. [6] have reported that TNF-*α* inhibits IRS-1 expression and tyrosyl phosphorylation. Since TSH can stimulate the production of TNF-*α*, we next tested whether TSH can suppress the protein expression and tyrosine phosphorylation of IRS-1. IRS-1 levels in adipocytes were quantified by Western blotting, and tyrosine phosphorylation was measured by immunoprecipitation after pretreatment with 100 nM insulin for 10 min and subsequent treatment with different concentrations of TSH (0.01, 0.1, and 1 mIU/ml) for 24 h. Tyrosine phosphorylation of IRS-1 decreased with increasing doses of TSH (*P* < 0.01 or *P* < 0.05) (Figure 4) and was inhibited by H89 and BAY 11-7082 (Figures 5(a) and 5(b)). The opposite effects were observed for TSH-dependent secretion of TNF-*α* and activation of NF-*κ*B DNA-binding activity.

**Figure 6:**
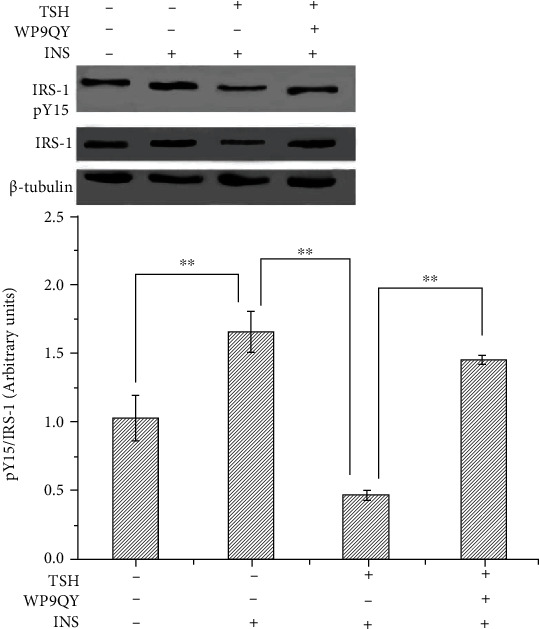
Effect of TSH on TNF-*α*-induced inhibition of insulin signals in 3T3-L1 adipocytes. Adipocytes were treated with 100 nM insulin and then coincubated with 1 mIU/ml TSH in the absence or presence of WP9QY. (a) Cells treated with insulin (the positive control group) exhibited higher IRS-1 tyrosyl phosphorylation levels than untreated control cells (the negative control group). TSH decreased IRS-1 tyrosyl phosphorylation. (b) Pretreatment with WP9QY significantly reversed the TSH-induced decrease in IRS-1 tyrosyl phosphorylation. The data are presented as the mean ± SD (*n* = 3) (^∗∗^*P* < 0.01).

IRS-1 is the major insulin-like growth factor-I receptor (IGF-IR). Insulin resistance is associated with a reduced ability of insulin to activate a variety of events in the insulin signaling system, such as tyrosine phosphorylation of IRS-1 [14]. It has been reported that TNF-*α* is the key mediator of insulin resistance because it can reduce insulin signaling by potently inhibiting insulin receptor (IR) tyrosine kinase, which might reduce the ability of IRS-1 to transduce signals in the insulin signaling system [5, 14]. Next, we aimed to identify the underlying molecular mechanisms responsible for TSH-mediated inhibition of IRS-1 tyrosyl phosphorylation (Tyr^15^). We investigated the effects of TSH on IRS-1 tyrosyl phosphorylation in adipocytes treated with 100 nM insulin and then coincubated with 1 mIU/ml TSH in the absence or presence of WP9QY. In the present study, cells treated with insulin (the positive control group) exhibited ~61% higher IRS-1 tyrosyl phosphorylation than the untreated control cells (Figure 6). IRS-1 tyrosyl phosphorylation was ~72% lower in TSH-treated 3T3-L1 adipocytes than in positive control cells (insulin-treated cells). WP9QY is a TNF-*α* antagonist designed to mimic the most critical tumor necrosis factor (TNF) recognition loop on TNF receptor I. It prevents interactions of TNF with its receptor. Pretreatment with WP9QY significantly reversed the TSH-induced decrease in IRS-1 tyrosyl phosphorylation (Figure 6). Taken together, these observations strongly suggest that TSH plays an important role in downregulating IRS-1 tyrosyl phosphorylation by inducing TNF-*α* secretion in 3T3-L1 adipocytes.

## 4. Discussion

As dietary structures and lifestyles change, the incidence and prevalence of T2DM continue to increase. In 2010, there were approximately 220 million DM patients worldwide, and the rates of T2DM are expected to reach epidemic levels before 2030 [1]. The increases in incidence and prevalence will be associated with significant increases in the numbers of patients with diabetes complications, which could lead to disastrous outcomes. Insulin resistance is a characteristic feature in most patients with T2DM.

Insulin is a pleiotropic hormone that has diverse functions; for example, it stimulates nutrient transport into cells, regulates glycometabolism, modifies enzymatic activity, and regulates energy homeostasis via actions in the arcuate nucleus [15, 16]. Reduced insulin action is associated with reduced insulin-stimulated activity of enzymes such as glycogen synthase and hexokinase [17, 18], and adipose tissue insulin resistance (adipo-IR) plays important roles [19, 20]. Adipose tissue functions not only as an energy depot [21] but also as an organ that exerts its effects through both paracrine and endocrine mechanisms [22]. Adipose tissue is understood to secrete numerous cytokines (adipokines), such as leptin [2], TNF-*α*, and IL-6 [3, 4]. It has been reported that these adipokines are the key mediators of insulin resistance because they suppress insulin activity (especially TNF-*α*) [5].

Recently, increasing numbers of studies have focused on the correlation between TSH and insulin resistance. An elevated risk for insulin resistance appears to be independently associated with subclinical hypothyroidism [23]. In addition, numerous reports have suggested that serum TSH levels are significantly associated with the homeostasis model assessment index for insulin resistance (HOMA-IR) [10] and negatively associated with the insulin sensitivity index [12, 23, 24]. Moreover, high-normal TSH levels are significantly associated with increased insulin resistance [10]. Currently, controversy exists regarding the upper limit of normal serum TSH values (above which treatment should be indicated), and it has been suggested that baseline thyrotropin concentrations greater than 2.2 mIU/L may be predictive of hypothyroidism in patients with DM [11]. In a previous study, we found that serum levels of TSH were higher in T2DM patients than in nondiabetic volunteers. Overall, there is an abundance of evidence that TSH levels are associated with insulin resistance, although the mechanisms of this association are unknown.

Many studies have shown that TSHR is expressed in thyrocytes as well as in extrathyroidal cells [7, 8]. The extrathyroidal effects of TSH on adipocytes have also been extensively studied [25, 26]. TSH, which is secreted by the anterior pituitary gland, not only regulates the endocrine function of the thyroid gland but also acts on adipocytes by binding directly to TSH receptors (TSHRs) expressed on adipocytes [7, 27–29]. Lisboa et al. [30] reported that TSH stimulates leptin secretion by adipocytes. It has also been reported that TSH stimulates 3T3-L1 adipocytes to release cAMP and glycerin in a dose-dependent manner [31]. We have reported previously that TSH stimulates TNF-*α* secretion from adipocytes via a cAMP-PKA-dependent pathway [9]. Likewise, in our present study, we found that TSH increased NF-*κ*B DNA-binding activity in adipocytes and that TNF-*α* secretion increased with increasing doses of TSH. However, all of these effects were alleviated by BAY 11-7082 (a nuclear NF-*κ*B inhibitor) and H89 (an inhibitor of cAMP-dependent PKA). NF-*κ*B is a multidirectional transcription factor that is involved in regulating the transcription of inflammatory mediators, cytokines, and growth factors, among other molecules. Transcriptional activation of NF-*κ*B increases the mRNA expression of NF-*κ*B target genes such as TNF*α*. Our data demonstrate that TSH-stimulated NF-*κ*B DNA-binding activity results in enhanced TNF-*α* transcriptional activity via the PKA signaling pathway.

The functions of insulin are exerted across a variety of insulin target tissues through several intracellular signaling cascades. Under normal conditions, insulin binds to IR in cell membranes to activate IR tyrosine kinase and triggers a series of insulin signal transduction responses in cells. The insulin signaling cascade branches into two main pathways. The first is the phosphatidylinositol 3-kinase- (PI3K-) AKT pathway, which is largely responsible for the effect of insulin on glucose uptake as well as other metabolic actions of insulin, including suppression of gluconeogenesis. The second pathway is the Ras-mitogen-activated protein kinase (MAPK) pathway, which mediates gene expression [32]. IRS-1 is a cellular signaling carrier protein involved in the modulation of intracellular bioinformation and recognizes messages from insulin receptors. Insulin binding induces receptor tyrosine autophosphorylation, which is followed by the tyrosine phosphorylation of IRS-1 and IRS-2 [33]. Tyrosyl phosphorylation of IRS-1 and IRS-2 activates downstream molecules and subsequently modulates blood glucose levels. It has been suggested that IRS-1 functions in glycometabolism by regulating insulin signals in the muscle and adipose tissues, whereas IRS-2 is a major participant in hepatic insulin action [34]. IRS-1 is the main IGF-IR, as defects in insulin signaling typically involve this insulin receptor substrate. Notably, IRS-1-deficient mice show a phenotype of peripheral insulin resistance (mainly in muscle and white adipose tissue) [35, 36]. Tyrosine phosphorylation of IRS-1 initiates signal transduction. However, the serine/threonine phosphorylation of IRS-1 subsequently suppresses tyrosine phosphorylation and blocks insulin signaling [37].

Proinflammatory cytokines can cause insulin resistance in adipose, skeletal muscle, and liver tissue by inhibiting insulin signal transduction. The sources of cytokines in insulin-resistant states are the insulin target tissue themselves. TNF-*α* is a proinflammatory cytokine secreted partly by adipocytes. TNF-*α* is an important mediator of insulin resistance. The possible mechanisms by which TNF-*α* impairs insulin signal transduction involve downregulation of IR and IRS-1 expression, inhibition of tyrosyl phosphorylation of IR and IRS-1, increased serine/threonine phosphorylation of IRS-1, decreased activity of IR kinase and protein tyrosine phosphatases (PTPs), and inhibition of insulin-stimulated glucose transporters [38, 39]. A previous study has revealed that treatment of cultured 3T3-L1 adipocytes with TNF-*α* leads to reduced expression of the IR, IRS1, and glucose transporter 4 (GLUT4) genes as well as a decrease in insulin-stimulated glucose uptake. [40]. The initiating factors of this inflammatory response remain to be fully determined. Our current results demonstrate that treatment of cultured adipocytes with TSH inhibited insulin-stimulated IRS-1 tyrosyl phosphorylation. Pretreatment with BAY 11-7082 or H89 significantly reversed the TSH-induced decrease in IRS-1 tyrosyl phosphorylation. The opposite effects were observed for TSH-dependent secretion of TNF-*α* and activation of NF-*κ*B DNA-binding activity. WP9QY, a TNF-*α* antagonist, was designed to mimic the most critical TNF recognition loop on TNF receptor I. It prevents interactions of TNF with its receptor. Pretreatment with WP9QY prevented the TSH-induced decreased in IRS-1 expression and tyrosyl phosphorylation in 3T3-L1 adipocytes. The present results demonstrate that TSH can inhibit IRS-1 tyrosyl phosphorylation in adipocytes by stimulating the production of TNF-*α* via TSH-mediated promotion of NF-*κ*B DNA-binding activity.

Downregulation of IRS-1 expression and tyrosyl phosphorylation suppresses the activity of PI3K. Suppression of PI3K activity might reduce insulin sensitivity and the efficiency of translocation of glucose transporters, leading to inhibition of glycogen synthesis-related gene modulation [41]. On the basis of our previous results indicating that TSH can downregulate GLUT4 expression and inhibit GLUT4 translocation to the plasma membrane [9], we speculate that TSH might play a pivotal role in the development of insulin resistance and that TSH is an important therapeutic target for improvement of insulin resistance.

Currently, the biological effects of TSH on adipocytes are likely underappreciated. The novelty of our study resides in the identification of TSH as a potentially novel mediator of the development of insulin resistance in adipocytes. However, the mechanism of insulin resistance is complicated. The results of this study are limited to in vitro cultures of 3T3-L1 adipocytes and cannot fully explain the role of TSH in human adipose tissue. Further studies on this topic may be warranted.

## Data Availability

The analyzed datasets generated during the study are available from the corresponding author on reasonable request.
